# Top-down proteomic identification of plasmid and host proteins produced by pathogenic *Escherichia coli* using MALDI-TOF-TOF tandem mass spectrometry

**DOI:** 10.1371/journal.pone.0260650

**Published:** 2021-11-29

**Authors:** Clifton K. Fagerquist, Claire E. Dodd

**Affiliations:** Produce Safety & Microbiology, Western Regional Research Center, Agricultural Research Service, U.S. Department of Agriculture, Albany, California, United States of America; Fisheries and Oceans Canada, CANADA

## Abstract

Fourteen proteins produced by three pathogenic *Escherichia coli* strains were identified using antibiotic induction, MALDI-TOF-TOF tandem mass spectrometry (MS/MS) and top-down proteomic analysis using software developed in-house. Host proteins as well as plasmid proteins were identified. Mature, intact protein ions were fragmented by post-source decay (PSD), and prominent fragment ions resulted from the aspartic acid effect fragmentation mechanism wherein polypeptide backbone cleavage (PBC) occurs on the C-terminal side of aspartic acid (D), glutamic acid (E) and asparagine (N) residues. These highly specific MS/MS-PSD fragment ions were compared to b- and y-type fragment ions on the C-terminal side of D-, E- and N-residues of *in silico* protein sequences derived from whole genome sequencing. Nine proteins were found to be post-translationally modified with either removal of an N-terminal methionine or a signal peptide. The protein sequence truncation algorithm of our software correctly identified all full and truncated protein sequences. Truncated sequences were compared to those predicted by SignalP. Nearly complete concurrence was obtained except for one protein where SignalP mis-identified the cleavage site by one residue. Two proteins had intramolecular disulfide bonds that were inferred by the *absence* of PBC on the C-terminal side of a D-residue located within the disulfide loop. These results demonstrate the utility of MALDI-TOF-TOF for identification of full and truncated bacterial proteins.

## Introduction

Identification and characterization of potentially pathogenic microorganisms requires technology and protocols that are simple, rapid, highly specific, and robust. Matrix-assisted laser desorption/ionization time-of-flight mass spectrometry (MALDI-TOF-MS) has found a niche application in the taxonomic identification of bacteria by generating a MS protein profile or “fingerprint” [1]. Typically, genus and species are readily identified by this technique although there have been some reports of higher taxonomic resolution [2]. The popularity of this approach is due to a relatively simple sample preparation, rapid analysis time and the fact that personnel, not formally trained in mass spectrometry, can operate such a platform, and perform data analysis with the pattern recognition software specifically designed for bacterial identification. The success of this approach led to further advances of this technology for top-down proteomic identification of specific proteins and virulence factors of pathogenic microorganisms [3–5].

Top-down proteomic analysis is the determination of the mass, sequence, and post-translational modifications (PTMs) of a mature intact protein without prior proteolytic digestion [6]. Top-down analysis has largely been the domain of high resolution (HR) and high accuracy mass analyzers, e.g. Orbitrap, as they are sensitive and can distinguish slight differences in protein and fragment ion mass-to-charge (*m/z*). Electrospray ionization (ESI) has been the ionization technique of choice for top-down because it results in multiply charged peptide and protein ions which bring the *m/z* of their discrete charge states below the upper bound of most quadrupole and trapping mass analyzers, i.e. *m/z* 3000. However, the multiply charged nature of ESI means that a sample comprised of a mixture of proteins must be separated chromatographically, either by off-line or on-line high performance liquid chromatography (HPLC), prior to ionization to reduce MS spectral complexity that would otherwise be too congested for MS deconvolution and charge state isolation for tandem mass spectrometry (MS/MS) due to overlapping charge state envelopes of multiple proteins. LC separation also reduces competitive ionization between analytes that would occur with an unfractionated mixture of proteins analyzed by ESI.

Several research groups have successfully developed and utilized LC-ESI-HR-MS and MS/MS for protein profiling of bacterial proteins as well as targeted and/or untargeted top-down identification of bacterial protein ions [7–13]. Protein profiling by LC-ESI-HR-MS involves deconvoluting and deisotoping the charge state envelopes of eluting proteins across a 1–2 hr chromatographic analysis resulting in a reconstructed MS profile of protein masses and their intensities. Such a MS protein profile is similar in appearance to a MALDI-TOF-MS spectrum but with many more proteins being detected and often with higher molecular weights which are useful in discriminating between closely related bacterial strains. Top-down identification of these bacterial proteins by MS/MS has revealed the complexity of PTMs that contribute to the proteoform of a microorganism [7, 8, 10].

Although LC-ESI-HR-MS and MS/MS is the platform of choice for top-down because of its higher resolution/accuracy and the arsenal of dissociation techniques that have been developed for its use [6, 14, 15], these platforms require significant skill and expertise to operate and maintain. In addition, technical replicates involving LC separation are time-consuming and labor intensive with complicated data analysis (deconvolution and deisotoping) after data acquisition. Alternatively, a few research groups have pursued MALDI tandem-TOF (TOF-TOF) for top-down proteomic analysis to identify low charge state intact protein ions from unfractionated bacterial cell lysates or LC-fractionated lysates [3–5]. MALDI-TOF-TOF has certain advantages because of its speed, ease-of-use, and simplicity which is especially relevant for microorganism identification and characterization by personnel not formally trained in mass spectrometry. However, one of the limitations of MALDI-TOF-TOF is the restricted number of dissociation techniques available. Only in-source decay (ISD) [16], high energy collision-induced dissociation (HE-CID) [17, 18], and post-source decay (PSD) [3, 4, 19, 20] have been successfully integrated with MALDI-TOF and TOF-TOF platforms. These dissociation techniques have their advantages and disadvantages. ISD (also referred to as T3-sequencing) can provide amino acid sequencing from N- and C-termini of a protein but is only compatible with pure protein samples [16, 21]. Analysis of protein mixtures by ISD is problematical because of the ambiguity in attributing specific fragment ions to specific precursor ions because the precursor ion is not isolated prior to fragmentation. The direct bond cleavage possible with HE-CID (1–2 keV) is most efficient when applied to peptide ions but less effective for protein ions. PSD has proved effective at fragmentation of proteins under 20 kDa but cleavage of the polypeptide backbone involves an ergodic molecular ion rearrangement involving the side-chain of specific residues: aspartic acid (D), glutamic acid (E), and/or asparagine (N). This fragmentation mechanism is called the *aspartic acid effect* and occurs on the C-terminal side of D-, E- and N-residues [20, 22–24]. Although the number of sequence-specific fragment ions is limited, PSD has the advantage of being compatible with MS/MS allowing isolation of the precursor ion prior to fragmentation and thus the ability to attribute specific fragment ions to a specific protein precursor ion which is critical when analyzing a mixture of proteins, e.g. bacterial cell lysate.

Analysis of bacterial pathogens is particularly relevant for this technology as rapid identification and characterization is often required to better inform appropriate treatment of time-sensitive bacterial infections and/or address a public health crisis [1, 2, 25]. Bacterial pathogen characterization invariably focuses on issues of antibiotic resistance and/or specific virulence factors that the bacteria may produce under certain conditions. In that regard, bacteriophages and plasmids often play a prominent role as they may carry antimicrobial resistance (AMR) or bactericidal genes that increase host survivability and/or toxin genes that increase host virulence. In addition, phages and plasmids represent mobile genetic elements which are vectors of horizontal gene transfer leading to the spread of bacterial virulence and AMR [1, 2].

Previously, we have identified different types and subtypes of Shiga toxin (Stx) from Shiga toxin-producing *E*. *coli* (STEC) using antibiotic induction, MALDI-TOF-TOF-MS/MS and top-down proteomic analysis [26, 27]. This approach has been quite successful in Stx identification as well as demonstrating the strain-dependent nature of Stx production across STEC strains. In the current work, we have extended this analysis to identify host and plasmid proteins produced by three pathogenic *E*. *coli* strains using antibiotic induction, MALDI-TOF-TOF-MS/MS-PSD and top-down proteomic analysis with software developed in-house recently. We observe a significant shift in the proteins detected as a function of antibiotic concentration. Antibiotic exposure appears to trigger the production of cold-shock proteins, a carbon utilization protein as well as plasmid proteins including bactericidal immunity proteins. Portions of this work were presented at the *2021 ASM Microbe-World Microbe Forum* [28].

## Material & methods

### Microbiology

Three pathogenic *E*. *coli* strains were analyzed: *E*. *coli* O113:H21 strain RM7806 [29], *E*. *coli* O113:H21 strain RM7807 [29], and *E*. *coli* O104:H4 strain RM14735 [30, 31]. The protocol has been described in detail previously [32]. Briefly, bacterial strains were cultured overnight at 37 °C on Luria-Bertani agar (LBA) supplemented with either mitomycin-C (MMC) or ciprofloxacin (Cip). Cells were harvested using a 1 μL sterile loop. Two μL of cells were transferred to an O-ring lined screw-top 2 μL microcentrifuge tube containing 300 μL of water. The tube was capped, vortexed briefly and centrifuged at 13,000 rpm for two minutes.

### Mass spectrometry

MS (linear-mode) data and MS/MS (reflectron-mode) data are collected as separate data acquisitions on the 4800 MALDI-TOF-TOF mass spectrometer (*Sciex*). A 0.75 μL aliquot of sample supernatant was spotted onto a 384-stainless steel MALDI target plate and allowed to dry. The dried sample spot was then overlayed with a 0.75 μL aliquot of a saturated solution of sinapinic acid (Thermo Scientific) dissolved in a mixture of water, acetonitrile and trifluoracetic acid in proportions 67%, 33% and 0.2%, respectively. Dried MALDI sample spots were analyzed on the MALDI-TOF-TOF instrument which is equipped with a 200 Hz, 355 nm pulsed solid-state laser. MS data was collected in MS linear-mode. The instrument was externally calibrated for MS analysis using the +1 and +2 charge states of protein calibrants (cytochrome-C, lysozyme and myoglobin). One thousand laser shots were acquired and summed for each sample spot. Laser fluence was 5200 to 6000 (a.u.). Ions were accelerated from the source at 20.0 kV and detected by the linear detector. Raw MS data was viewed using *Data Explorer* software (Sciex) and was unprocessed.

MS/MS data was collected in reflectron-mode. The instrument was externally calibrated for MS/MS using the fragment ions of alkylated thioredoxin whose metastable precursor ion fragments efficiently by post-source decay (PSD) [33]. The *m/z* of the target protein (as measured by MS linear-mode) is manually entered into the software as the precursor ion to be isolated for MS/MS. Protein precursor ions were mass selected/isolated by the timed ion selector (TIS) on the basis of their arrival time at the TIS. The TIS window was narrowed to eliminate fragment ion spillover from adjacent protein ions. After precursor ion fragmentation in the collision cell (no collision gas), any *unfragmented* precursor ion is excluded by the metastable suppressor in order not to saturate the gain of the MS/MS reflectron detector. Ten thousand laser shots were acquired and summed for each protein ion analysis. MS/MS laser fluence was 6500–7000 (a.u.). At this higher laser fluence, a sample/matrix spot is exhausted upon experiment completion. Raw MS/MS data was viewed and processed using *Data Explorer* software in the following sequence: advanced baseline correction (peak width: 32; flexibility: 0.5; degree: 0.0), noise removal (2 standard deviations) and Gaussian smoothing (filter width: 31 points).

### Top-down proteomic analysis

**Fig 1:**
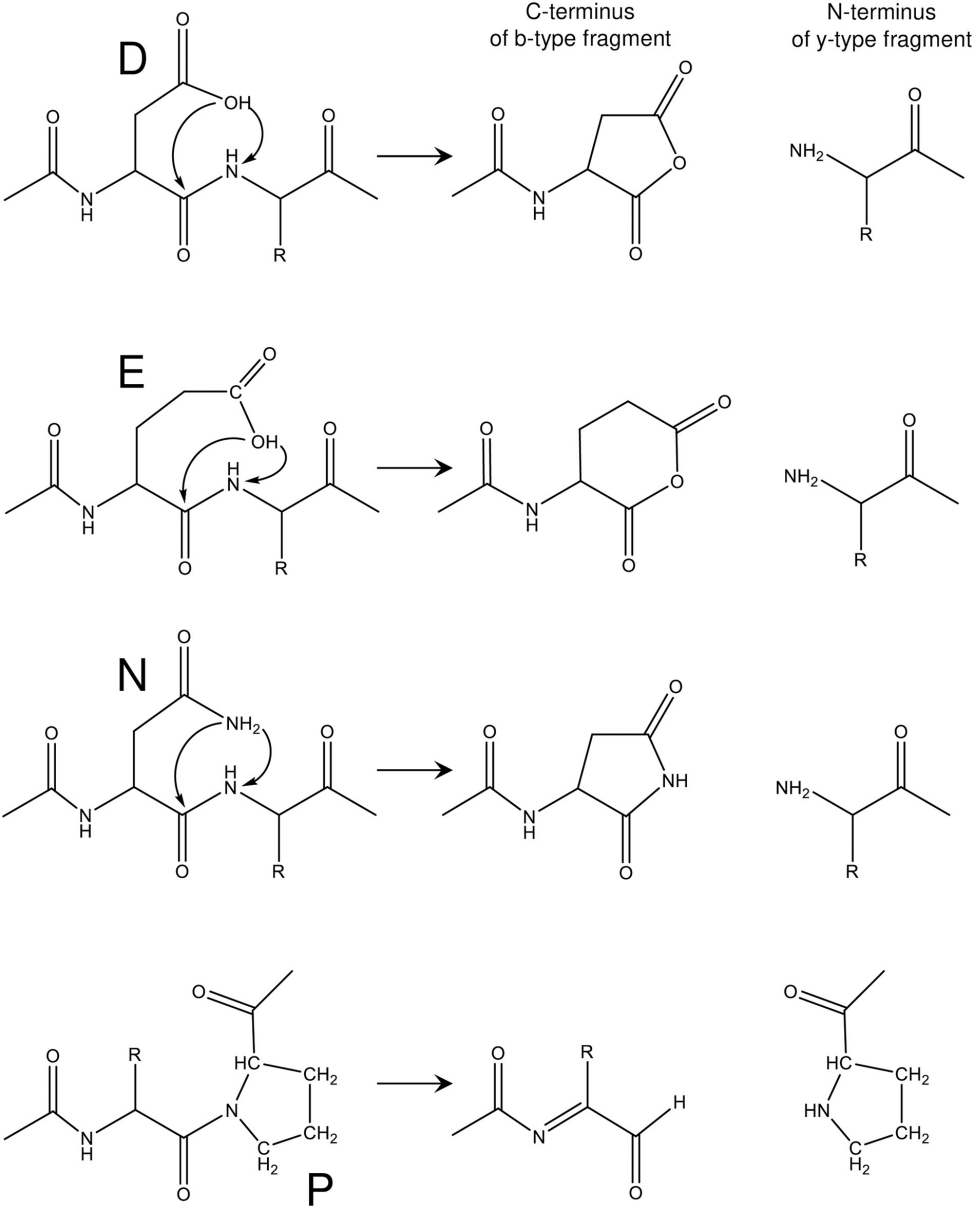
Schematic of the *aspartic acid effect* fragmentation mechanism of the polypeptide backbone cleavage on the C-terminal side of aspartic acid (D), glutamic acid (E) and asparagine (N) residues and the N-terminal side of proline (P) residues resulting in the formation of complementary b-type and y-type fragments. Whether a fragment is neutral or ionized depends on which basic residue of the protein precursor ion is sequestering the ionizing proton.

The software and its operation have been described in detail previously [34, 35]. Briefly, the measured mass (and mass tolerance) and the *m/z* of two (or more) prominent non-complementary fragment ions (and *m/z* tolerance) are entered into the software. The operator selects whether *in silico* protein sequence truncation is to be unrestricted (all protein fragments of a sequence are to be considered) or limited with respect to the maximum possible length N-terminal signal peptides. The operator then selects which, among four residues, result in polypeptide backbone cleavage (PBC), i.e. C-terminal side of D- and/or E- and/or N-residues and/or N-terminal side of P-residue. Fig 1 shows the aspartic acid effect for polypeptide backbone cleavage on the C-terminal side of D-, E- and N-residues and the N-terminal side of P-residues resulting in complementary b- and y-type fragments.

Whether a fragment is neutral or ionized depends on which basic residue of the protein precursor ion sequesters the ionizing proton. No other residue choices are allowed because prominent fragment ions result from PBC at only these residues. Fragment ions selected for comparison are the result of only PBC and do not include small neutral losses, e.g. -17 Da (loss of NH_3_) or -18 Da (loss of H_2_O) as the software does not consider these *in silico* fragment ions in the identification process. The maximum number of fragment ions to be matched is selected which is equal to or less than the number of fragment ions entered. Finally, a file containing *in silico* protein sequences (derived from whole genome sequencing) of the specific bacterial strain is downloaded from *National Center for Biotechnology Information* (NCBI), compiled into a single file, and selected for scanning. A pop-up window confirms the search parameters after which the user initiates the search. Within a few seconds, possible candidate sequences are displayed by another pop-up window. The user must manually confirm the correctness of any candidate sequence by comparing the *m/z* of major and minor fragment ions of the MS/MS data to *in silico* fragment ions generated using the candidate sequence and any mass spectrometry/proteomic software, e.g. GPMAW (Lighthouse data) [36].

## Results and discussion

### *E*. *coli* O113:H21 strain RM7806

**Fig 2:**
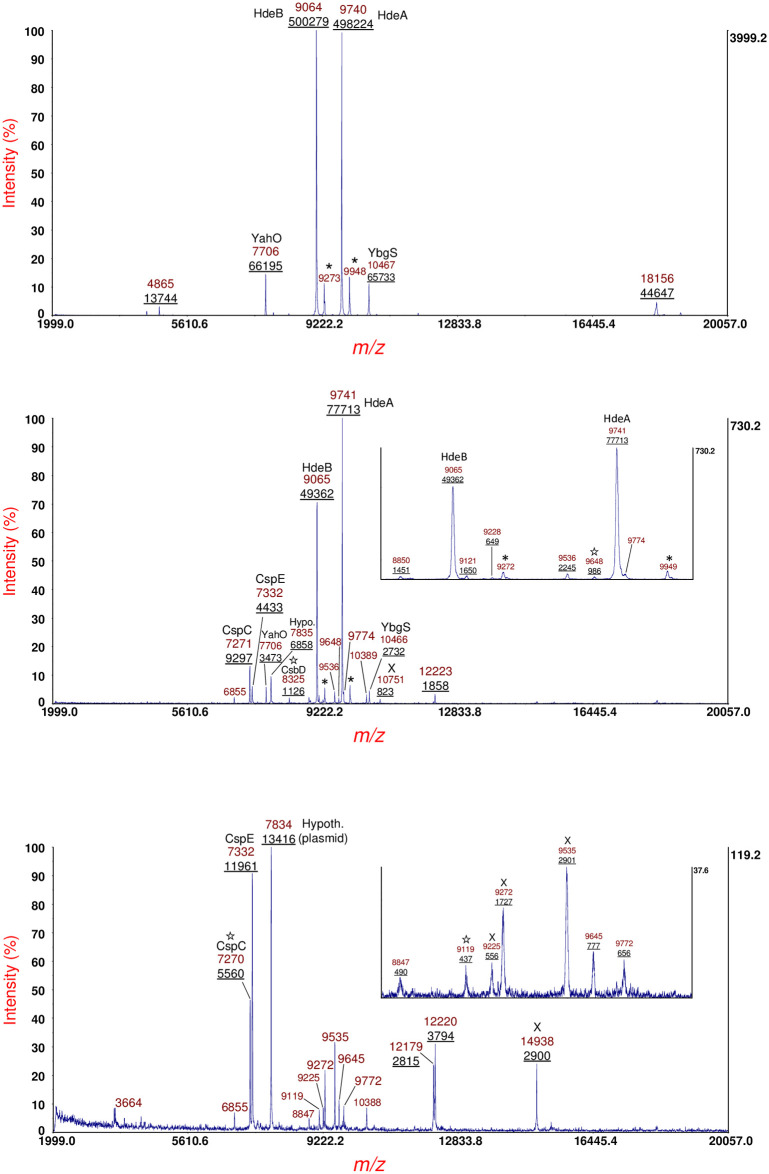
Top panel: MS of sample supernatant of *E*. *coli* O113:H21 strain RM7806 cultured overnight on LBA. Middle panel: MS of sample supernatant of strain cultured overnight on LBA supplemented with 400 ng/mL of MMC. Bottom panel: MS of sample supernatant of strain cultured overnight on LBA supplemented 600 ng/mL of MMC. Prominent protein ions are identified by the mass-to-charge (*m/z*) ratio. Integrated peak areas (underlined) are provided below the *m/z*. Expanded *m/z* are provided by inserts. Conserved proteins ions are labeled on the basis of previous identifications in other *E*. *coli* strains. Protein ions marked with an asterisk are MALDI matrix adducts. Protein ions marked with a star were analyzed by MS/MS in the current work. Protein ions marked with an X were analyzed by MS/MS, but the data was not of sufficient quality for an identification.

Fig 2 shows MS data of sample supernatant of *E*. *coli* O113:H21 strain RM7806 cultured overnight on LBA (top panel), LBA supplemented with 400 ng/mL of MMC (middle panel) and LBA supplemented 600 ng/mL of MMC (bottom panel). Prominent protein ions are identified by their mass-to-charge (*m/z*) ratio. Integrated peak areas (underlined) are provided below the *m/z*. Inserts of expanded *m/z* are shown in the middle and bottom panels. As can be seen, protein ion intensity varies with antibiotic concentration. Some highly conserved proteins ions are labeled on the basis of previous identifications in other *E*. *coli* strains [4]. Protein ions marked with an asterisk are MALDI matrix adducts [37]. Protein ions marked with a star were analyzed by MS/MS in the current work. Protein ions marked with an X were analyzed by MS/MS, but the data was not of sufficient quality for an identification.

**Fig 3:**
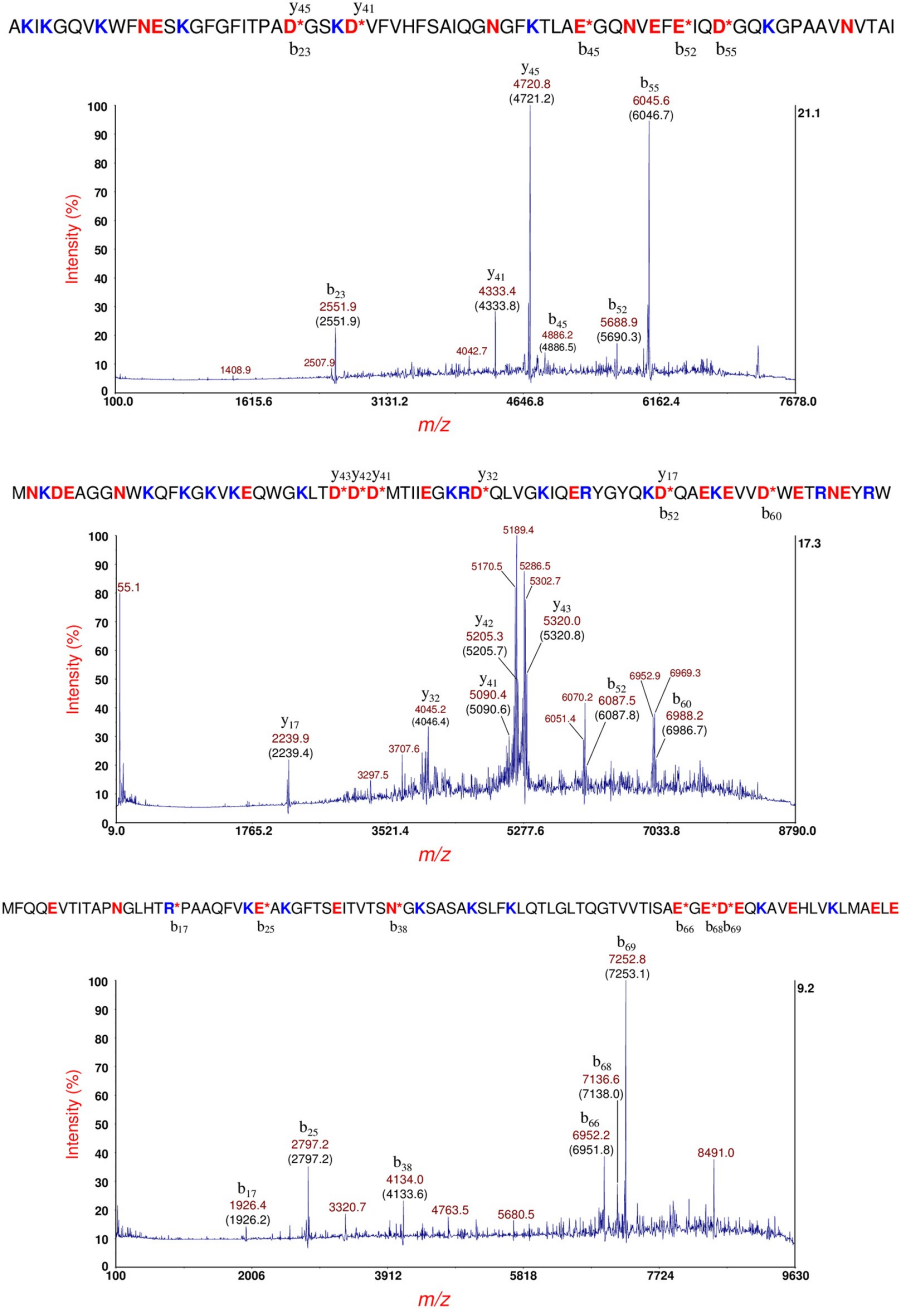
Top panel: MS/MS of the putative CspC at *m/z* 7270 [M+H]^+^ in Fig 2 (bottom panel). Prominent fragment ions are identified by their *m/z* and their fragment ion type/number (b- or y-type). The theoretical average *m/z* value is shown in parentheses. CspC protein sequence is provided above the spectrum. An asterisk (*) indicates the site of PBC. The resulting fragment ions are shown above and below the sequence. An asymmetric TIS window (-100/+30) was used to eliminate spillover of fragment ions from putative CspE at *m/z* 7332. Middle panel: MS/MS of the putative CsbD at *m/z* 8325 [M+H]^+^ (Fig 2, middle panel). Prominent fragment ions are identified. The average theoretical *m/z* is shown in parentheses. The CsbD sequence is provided above the MS/MS spectrum. Bottom panel: MS/MS of the protein ion at *m/z* 9119 [M+H]^+^ (Fig 1, bottom panel). The HPr sequence is shown above the MS/MS spectrum.

Fig 3 (top panel) shows MS/MS of the putative cold-shock protein C (CspC) at *m/z* 7270 [M+H]^+^ in Fig 2 (bottom panel). This protein ion also appears at *m/z* 7271 in Fig 2 (middle panel). Prominent fragment ions are identified by their *m/z* and their fragment ion type/number (b- or y-type) based on the protein identification using top-down proteomic software developed in-house [34, 35]. The theoretical average *m/z* value is shown in parentheses. Prominent fragment ions are the result of the *aspartic acid effect* wherein the polypeptide backbone fragments on the C-terminal side of aspartic acid (D), glutamic acid (E) [20, 22, 24], and asparagine (N) residues [38]. The identified protein sequence is provided above the spectrum. An asterisk (*) indicates the site of PBC. The resulting fragment ions are shown above and below the sequence. Based on MS and MS/MS data, the N-terminal methionine was removed in the mature CspC sequence consistent with the penultimate residue rule [39–41]. An asymmetric TIS window (-100/+30) was used to eliminate spillover of fragment ions from putative CspE at *m/z* 7332.

Fig 3 (middle panel) shows MS/MS of the putative cold-shock/stress-response protein: CsbD at *m/z* 8325 [M+H]^+^ (Fig 2, middle panel). A more complicated MS/MS spectrum is observed than that observed of CspC. Prominent fragment ions are identified. The theoretical average *m/z* value is shown in parentheses. Using our top-down software, the identified protein sequence is provided above the MS/MS spectrum. The greater complexity MS/MS of CsbD is due to small neutral dissociative losses (i.e. NH_3_ loss) from the side-chains of the four arginine residues (R36, R46, R64, R68) present in the sequence. Many of the most abundant fragment ions are the result of PBC *and* neutral losses (separated by multiples of ~17 Da). Because our software does not incorporate small neutral losses in the identification process, fragment ions resulting from *only* PBC were used for protein identification even though some of these fragment ions were not necessarily the most abundant.

Fig 3 (bottom panel) shows MS/MS of the protein ion at *m/z* 9119 [M+H]^+^ (Fig 2, bottom panel). This protein ion also appears at *m/z* 9121 in Fig 2 (middle panel, insert). The identified protein is that of the histidine-phosphorylatable phosphocarrier protein (HPr) whose sequence is shown above the MS/MS spectrum. This protein contains only one arginine residue (R17) and all fragment ions have R17 in their sequence suggesting that the ionizing proton is likely sequestered at this residue due to its higher gas phase basicity although there are seven other ionization sites in the sequence, i.e. lysine residues (K). The most abundant fragment ion (b_69_) is the result of PBC on the C-terminal side of the only aspartic acid residue in the protein sequence: D69. Facile PBC at D69 is a direct result of its short side-chain and its carboxylic acid functional group. Detection of HPr is interesting as it is a global regulator of carbon metabolism [42]. It is not clear why the HPr gene would be expressed because of exposure antibiotic exposure although it is possible that antibiotic stress may affect carbon metabolism. HPr may be phosphorylated at histidine (H15) and/or at serine (S46), however only the unphosphorylated HPr was detected. It is possible that the low pH conditions used for sample preparation and application of the MALDI matrix may reverse phosphorylation prior to analysis.

**Fig 4:**
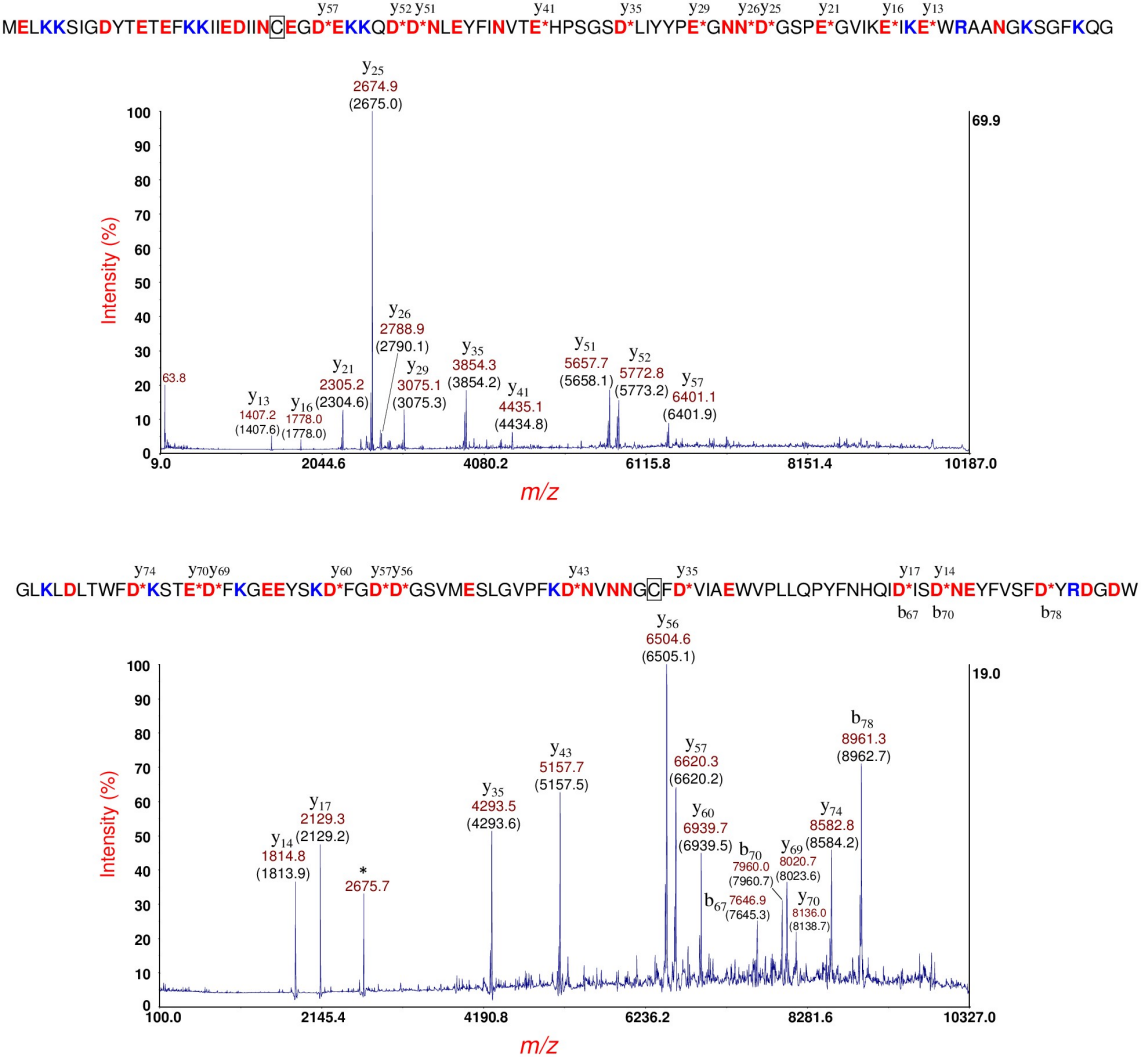
Top panel: MS/MS of the protein ion at *m/z* 9648 [M+H]^+^ in Fig 2 (middle panel). This protein ion was identified as the immunity protein for bacteriocin (ImBac) whose sequence is shown above the MS/MS spectrum. This protein has a single cysteine residue (boxed) which is assumed to be in its reduced state. Bottom panel: MS/MS of the protein ion at *m/z* 9779 [M+H]^+^ shown in S1 Fig. The protein was identified as the immunity protein for colicin E3 (Im3) bactericidal protein (shown above the MS/MS spectrum). This protein has a single cysteine residue (boxed) which is assumed to be in its reduced state. The fragment ion at *m/z* 2675.7 marked with an asterisk (*) is spillover from the most abundant fragment ion (y_25_) of ImBac (top panel).

Fig 4 (top panel) shows MS/MS of the protein ion at *m/z* 9648 [M+H]^+^ Fig 2 (middle panel). The protein ion also appears at *m/z* 9645 in Fig 2 (bottom panel). This protein ion was identified as the immunity protein for bacteriocin (ImBac) whose sequence is shown above the MS/MS spectrum [43]. ImBac is a plasmid-borne protein and has a single cysteine residue (boxed) which is assumed to be in its reduced state. ImBac has a single arginine residue (R74), and all fragment ions possess R74 which suggests that the ionizing charge/proton is sequestered at that residue.

Fig 4 (bottom panel) shows MS/MS of the protein ion at *m/z* 9779 [M+H]^+^ shown in S1 Fig. This protein ion also appears at *m/z* 9774 and 9772 in Fig 2 (middle and bottom panels, respectively) and was identified as the immunity protein for colicin E3 (Im3) another bactericidal protein [43, 44]. The Im3 gene is also a plasmid-encoded, and its protein sequence is shown above the MS/MS spectrum. This protein has a single cysteine residue (boxed) and is assumed to be in its reduced state. Using the simple formula of b (*m/z*) + y (*m/z*)– 2H^+^ (*m/z*) = protein mass (Da) and the *m/z* of the two CFIP: b_67_/y_17_ and b_70_/y_14_, it is possible to calculate the mass of the protein more accurately giving a value of 9773.5 ± 1.0 Da which is closer to its theoretical value of 9772.5 Da. Although Im3 also has a single arginine residue (R80), three b-type fragment ions do not have R80 in their sequence. In consequence, the location of the ionizing proton does not appear to be exclusive to R80. This lack of charge exclusivity may be due to R80 being adjacent to D81, and these adjacent residues may form a Zwitterion that inhibits sequestration of the ionizing charge at R80. Alternatively, R80 may be less accessible for ionization due to partial folding of the protein. The fragment ion at *m/z* 2675.7 marked with an asterisk (*) is spillover from the most abundant fragment ion (y_25_) of ImBac (top panel). A summary of identifications for *E*. *coli* O113:H21 strain RM7806 is provided in Table 1.

**Table 1:** Protein biomarker identification.

Strain	Protein	*m/z* (M+H]^+^ MS linear-mode	Theo. Ave MW (Da) of mature protein	Mature amino acid sequence	Post-translational modification (MS & MS/MS)	SignalP 5.0
**RM7806**	Cold-shock protein: CspC	7270	7271.1	AKIKGQVKWFNESKGFGFITPADGSKDVFVHFSAIQGNGFKTLAEGQNVEFEIQDGQKGPAAVNVTAI	Removal of N-terminal Met	N/A
"	Cold-shock protein: CbsD	8325	8325.2	MNKDEAGGNWKQFKGKVKEQWGKLTDDDMTIIEGKRDQLVGKIQERYGYQKDQAEKEVVDWETRNEYRW	None	N/A
"	Histidine-phosphorylatable phosphocarrier protein: HPr	9119	9119.3	MFQQEVTITAPNGLHTRPAAQFVKEAKGFTSEITVTSNGKSASAKSLFKLQTLGLTQGTVVTISAEGEDEQKAVEHLVKLMAELE	None	N/A
"	Immunity protein of bacteriocin: ImBac (plasmid)	9648	9645.5 (reduced)	MELKKSIGDYTETEFKKIIEDIIN**C**EGDEKKQDDNLEYFINVTEHPSGSDLIYYPEGNNDGSPEGVIKEIKEWRAANGKSGFKQG	None	N/A
"	Immunity protein of colicin E3: Im3 (plasmid)	9779	9772.5 (reduced)	GLKLDLTWFDKSTEDFKGEEYSKDFGDDGSVMESLGVPFKDNVNNG**C**FDVIAEWVPLLQPYFNHQIDISDNEYFVSFDYRDGDW	Removal of N-terminal Met	N/A
**RM7807**	Cold-shock protein: CspC	7272	7271.1	AKIKGQVKWFNESKGFGFITPADGSKDVFVHFSAIQGNGFKTLAEGQNVEFEIQDGQKGPAAVNVTAI	Removal of N-terminal Met	N/A
"	Cold-shock protein: CspE	7333	7332.2	SKIKGNVKWFNESKGFGFITPEDGSKDVFVHFSAIQTNGFKTLAEGQRVEFEITNGAKGPSAANVIAL	Removal of N-terminal Met	N/A
"	Immunity protein of bacteriocin: ImBac (plasmid)	9648	9645.5 (reduced)	MELKKSIGDYTETEFKKIIEDIIN**C**EGDEKKQDDNLEYFINVTEHPSGSDLIYYPEGNNDGSPEGVIKEIKEWRAANGKSGFKQG	None	N/A
"	Immunity protein of colicin E3: Im3 (plasmid)	9778	9772.5 (reduced)	GLKLDLTWFDKSTEDFKGEEYSKDFGDDGSVMESLGVPFKDNVNNG**C**FDVIAEWVPLLQPYFNHQIDISDNEYFVSFDYRDGDW	Removal of N-terminal Met	N/A
"	Histidine-phosphorylatable phosphocarrier protein: HPr	9122	9119.3	MFQQEVTITAPNGLHTRPAAQFVKEAKGFTSEITVTSNGKSASAKSLFKLQTLGLTQGTVVTISAEGEDEQKAVEHLVKLMAELE	None	N/A
**RM14735**	YahO protein	7708	7706.5	AELMTKAEFEKVESQYEKIGDISTSNEMSTADAKEDLIKKADEKGADVLVLTSGQTDNKIHGTANIYKKK	Removal of 21-residue signal peptide	Signal peptide probability: 0.9716. Cleavage site probability: 0.7656
"	YbgS-like protein, AFS75454.1 hypothetical protein O3K_17905 [Escherichia coli O104:H4 str. 2011C-3493]	10469	10464.9 (oxidized)	ADSGAQTNNGQANAAADAGQVAPDARENVAPNNVDNNGVNTGSGGTMLHSDGSSMNNDGMTKDEEHKNTM**C**KDGR**C**PDINKKVQTGDGINNDVDTKTDGTTQ	Removal of 24-residue signal peptide. Disulfide bond inferred from MS/MS data.	Signal peptide probability: 0.9976. Cleavage site probability: 0.9832
"	Hypothetical protein C22711_3545, EGT69515.1, [Escherichia coli O104:H4 str. C227-11]	10892	10881.4	AEHSEMKMTDMSTSASSQEYMAGMKDMHDKMMAAVNESDPDKAFAKGMVAHHEGAIAMAETELKYGKDPKMRKLAQDIIKAQKGEIEQMNKWLDSQK	Removal of 19-residue signal peptide	Signal peptide probability: 0.9633. Cleavage site probability: 0.5566. Predicted cleavage site off by one residue.
"	Hypothetical protein O3K_26197 (plasmid), AFS77026.1, [Escherichia coli O104:H4 str. 2011C-3493]	14852 (MS/MS 14835.7 ± 1.4 Da)	14835.7 (oxidized)	ASQQTTQTIRLTVTND**C**PVTITTTPPQTVGVSSTTPIGFSAKVTTSDQ**C**IKAGAKVWLWGTGPANKWVLQHAKVAKQKYTLNPSIDGGADFVNQGTDAKIYKKLTSGNKFLNASVSVNPKTQVLIPGEYTMILHAAVDF	Removal of 28-residue signal peptide. Disulfide bond inferred from MS/MS data.	Signal peptide probability: 0.9322. Cleavage site probability: 0.9106.

### *E*. *coli* O113:H21 strain RM7807

**Fig 5:**
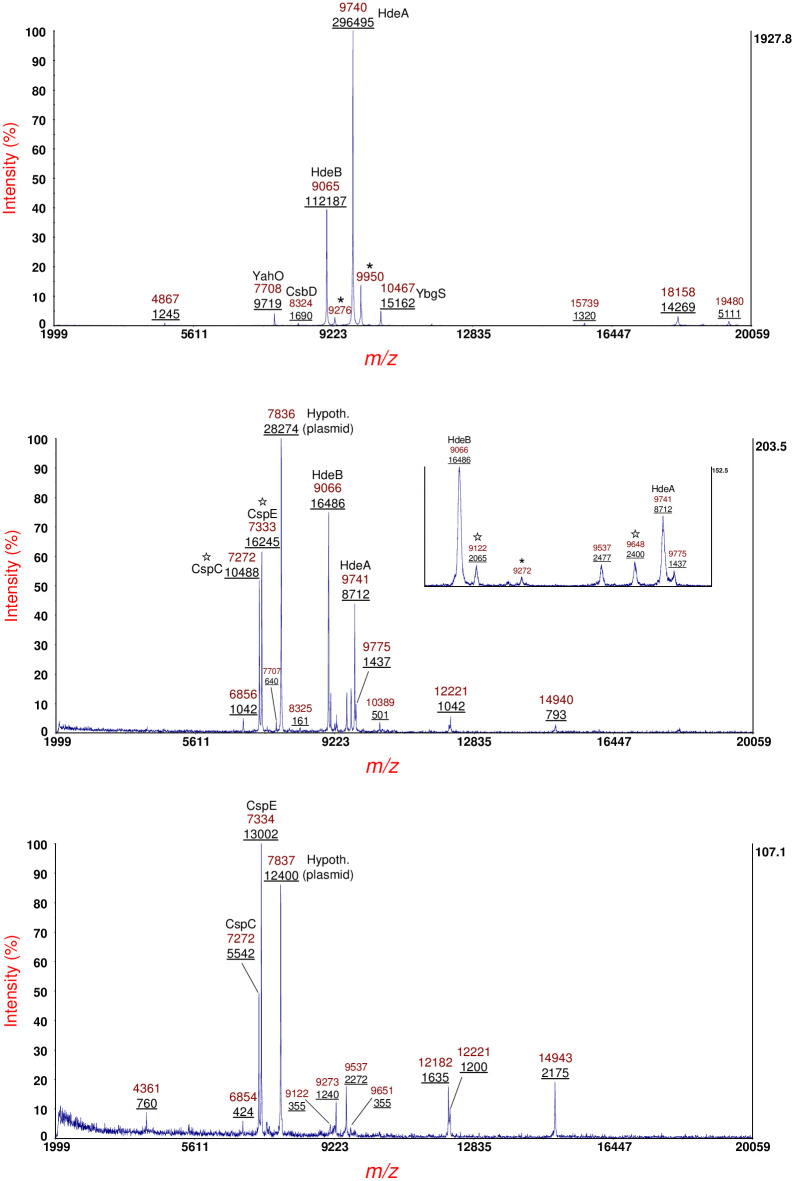
Top panel: MS of sample supernatant of *E*. *coli* O113:H21 strain RM7807 cultured overnight on LBA. Middle panel: MS of sample supernatant of strain cultured overnight on LBA supplemented with 400 ng/mL of MMC. An expanded *m/z* insert is shown. Bottom panel: MS of sample supernatant of strain cultured overnight on LBA supplemented 600 ng/mL of MMC. Prominent protein ions are identified by their *m/z*. Integrated peak areas (underlined) are provided below the *m/z*. Protein ions marked with an asterisk are matrix adducts. Protein ions marked with a star were analyzed by MS/MS. Conserved *E*. *coli* host proteins, e.g. acid stress proteins HdeA and HdeB, as well as YahO and YbgS, are labeled based on previous identifications.

Fig 5 shows MS data of sample supernatant of *E*. *coli* O113:H21 strain RM7807 cultured overnight on LBA (top panel), LBA supplemented with 400 ng/mL of MMC (middle panel) and LBA supplemented 600 ng/mL of MMC (bottom panel). Prominent protein ions are identified by their *m/z*. Integrated peak areas (underlined) are provided below the *m/z*. An expanded *m/z* insert is shown in the middle panel. Protein ions marked with an asterisk are matrix adducts. Protein ions marked with a star were analyzed by MS/MS. High copy, highly conserved host proteins, e.g. acid stress proteins HdeA and HdeB as well as YahO and YbgS, are assigned based on previous identifications (top panel). Upon culturing with LBA supplemented with 400 ng/mL MMC, putative cold-shock proteins CspC and CspE are detected along with a number of plasmid proteins. HdeA, HdeB and YahO are still detected but at lower abundance. Upon culturing with LBA supplemented with 600 ng/mL MMC, only CspC, CspE and plasmid proteins are detected.

**Fig 6:**
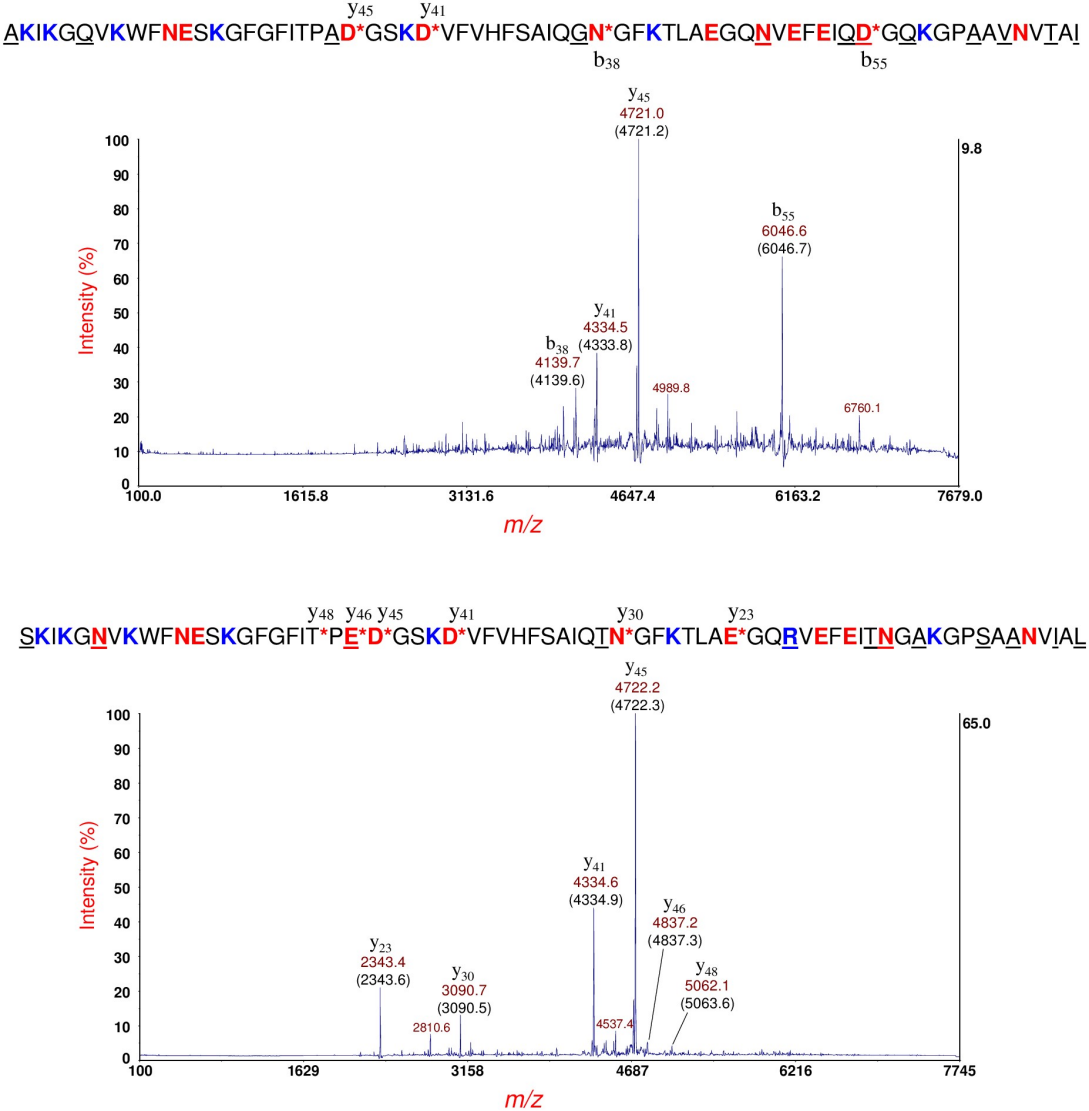
Top panel: MS/MS of the putative CspC at *m/z* 7272 [M+H]^+^ (Fig 4, middle panel). CspC sequence is shown above (without its N-terminal methionine) and underlined residues highlight differences in amino acid sequence between CspC and CspE (shown below). An asymmetric TIS window of -100/+30 was used to eliminate spillover of fragment ions of the putative metastable CspE ion. Bottom panel: MS/MS of the putative CspE at *m/z* 7333 [M+H]^+^ (Fig 4, middle panel). CspE sequence is shown above (without its N-terminal methionine). Underlined residues highlight differences in amino acid sequence between CspE and CspC (shown above). The TIS window was reversed (-30/+100) from its setting for CspC to eliminate fragment ion spillover of metastable CspC ion.

Fig 6 (top panel) shows MS/MS of the putative cold-shock protein C (CspC) at *m/z* 7272 [M+H]^+^ (Fig 5, middle panel). Based on MS and MS/MS data, the N-terminal methionine was removed in the mature CspC sequence consistent with the penultimate residue rule. An asymmetric TIS window of -100/+30 was used to eliminate spillover of fragment ions of the putative metastable CspE ion. Underlined residues highlight differences in amino acid sequence between CspC and cold-shock protein E (CspE) (shown below). Fig 6 (bottom panel) shows MS/MS of the putative CspE at *m/z* 7333 [M+H]^+^ (Fig 5, middle panel). The mature CspE sequence also has its N-terminal methionine removed. The asymmetry of the TIS window was reversed (-30/+100) to eliminate fragment ion spillover of metastable CspC ion. Interestingly, the *m/z* of y_45_ and y_41_ of CspC are different by 1 Th (1 *m/z* unit) from the *m/z* of y_45_ and y_41_ of CspE, respectively, although their fragment ion sequences are different by nine amino acid residues. For the more abundant y_45_ fragment ion of CspC and CspE, the slight mass difference is distinguishable. Another difference is that CspC has no R-residues whereas CspE has one (R48) and all the fragment ions generated by CspE include R48 in their fragment sequence. Finally, the precursor ion intensity of CspC and CspE are nearly of equal abundance (Fig 5, middle panel), but their fragmentation efficiencies are different. This is likely due to differences in denaturation of CspC vs. CspE prior to ionization. An unfolded protein is more likely to generate abundant fragment ions than a protein that is folded or partially unfolded.

**Fig 7:**
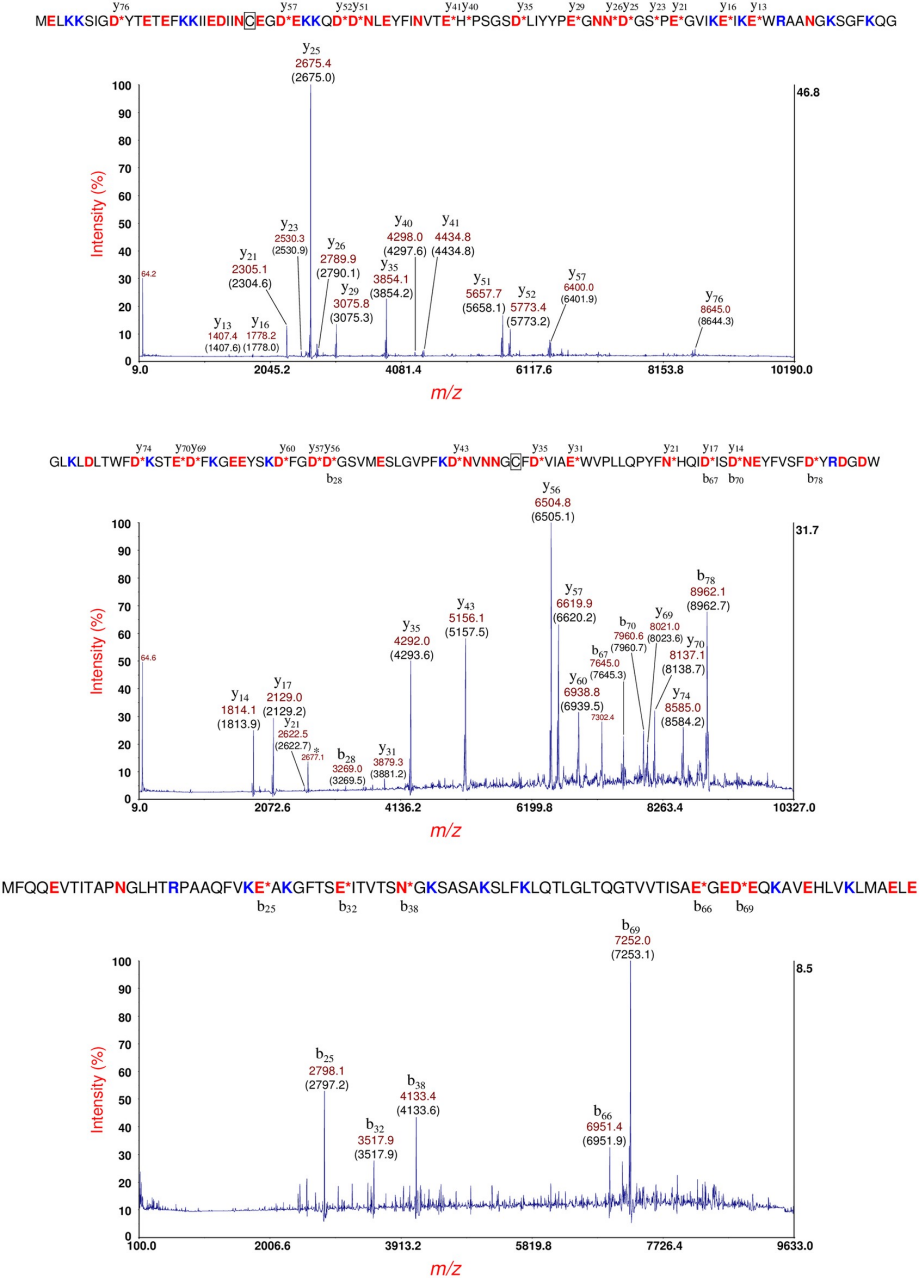
Top panel: MS/MS of the protein ion at *m/z* 9648 [M+H]^+^ shown in Fig 4 (middle panel). The protein sequence (shown above) is that of the immunity protein (ImBac) of bacteriocin, a plasmid-borne bactericidal enzyme and noted previously. A single cysteine residue (boxed) and is assumed to be in its reduced state. Middle panel: MS/MS of the protein ion at *m/z* 9778 [M+H]^+^ shown in S2 Fig. The protein was identified as the immunity protein (Im3) of the bactericidal enzyme: colicin E3 another plasmid-borne protein (sequence shown above). This protein also has one cysteine residue (boxed). Bottom panel: MS/MS of the protein ion at *m/z* 9122 [M+H]^+^ that appears in the middle and bottom panels of Fig 4. As with RM7806, this protein ion was identified as HPr (sequence shown above). An asymmetric TIS was used (-40/+80) to eliminate fragment ion spillover from metastable HdeB at *m/z* 9066.

Fig 7 (top panel) shows MS/MS of the protein ion at *m/z* 9648 [M+H]^+^ shown in Fig 5 (middle panel). This protein ion also appears at *m/z* 9651 in Fig 5 (bottom panel). The protein sequence (shown above) is that of ImBac noted previously. A single cysteine residue (boxed). Only y-type fragment ions are detected, and their sequences all have the only R-residue (R74) in the protein sequence suggesting that the ionizing proton is sequestered at R74. Fig 7 (middle panel) shows MS/MS of the protein ion at *m/z* 9778 [M+H]^+^ shown in S2 Fig. This protein ion also appears at *m/z* 9775 in Fig 5 (middle panel). The protein was identified as the immunity protein: Im3. The N-terminal methionine has been removed in the mature protein consistent with the penultimate rule and this protein also has one cysteine residue (boxed). Although Im3 has only one R-residue (R80), four b-type fragment ions do not have R80 in their sequence. In consequence, charge sequestration is predominantly (although exclusively) at R80. Three CFIP were also detected: b_28_/y_56_, b_67_/y_17_ and b_70_/y_14_, and used to calculate the protein mass: 9772.2 ± 0.5 Da which is within experimental error to its theoretical value of 9772.5 Da and closer to the measured mass in MS linear mode: 9777 Da. Fig 7 (bottom panel) shows MS/MS of the protein ion at *m/z* 9122 [M+H]^+^ that appears in the middle and bottom panels of Fig 5. As with RM7806, this protein ion was identified HPr (sequence shown above), and once again, only b-type ions were detected. All fragment ions contain the only R-residue (R17) in their sequence suggesting charge sequestration occurs primarily at R17. A narrower, asymmetric TIS was used (-40/+80) to eliminate fragment ion spillover from metastable HdeB at *m/z* 9066. A summary of identifications for *E*. *coli* O113:H21 strain RM7807 is provided in Table 1.

### *E*. *coli* O104:H4 strain RM14735

**Fig 8:**
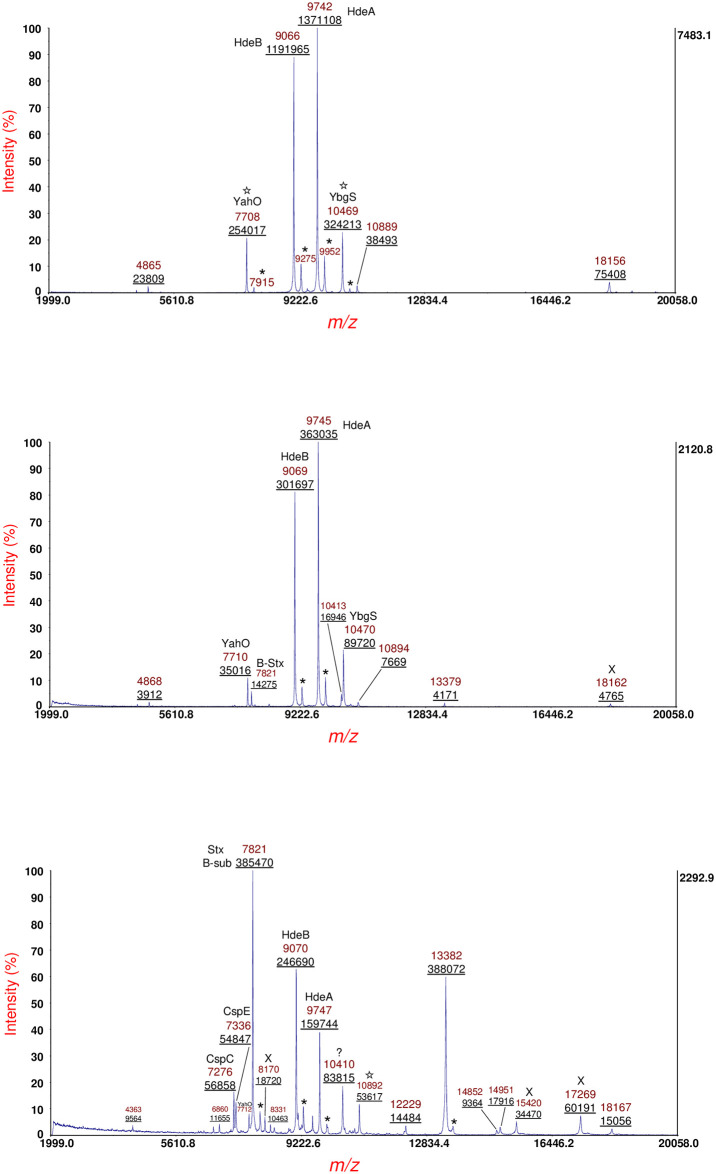
Top panel: MS of sample supernatant of *E*. *coli* O104:H4 strain RM14735 (German outbreak strain) cultured overnight on LBA. Middle panel: MS of sample supernatant of strain cultured overnight on LBA supplemented with 10 ng/mL of ciprofloxacin. Bottom panel: MS of sample supernatant of strain cultured overnight on LBA supplemented 20 ng/mL of ciprofloxacin. Prominent protein ions are identified by their *m/z* and in some cases assigned based upon previous identifications of conserved *E*. *coli* proteins. Integrated peak areas (underlined) are provided below the *m/z*. The B-subunit of Shiga toxin 2 is detected at *m/z* 7821 (middle and bottom panels) and had been previously identified for this strain. Protein ions marked with an asterisk are MALDI matrix adducts. Protein ions marked with a star were analyzed by MS/MS. Protein ions marked with a X were analyzed by MS/MS, but the data was not of sufficient quality for an identification. The protein ion at *m/z* 10410 (bottom panel) highlighted with a question mark resulted in quality MS/MS data (not shown) but was not able to be identified.

Fig 8 shows MS of sample supernatant of *E*. *coli* O104:H4 strain RM14735 (German outbreak strain [30, 31]) cultured overnight on LBA (top panel), LBA supplemented with 10 ng/mL of ciprofloxacin (middle panel) and LBA supplemented 20 ng/mL of ciprofloxacin (bottom panel). Prominent protein ions are identified by their *m/z* and in some cases assigned based upon previous identifications of abundant highly conserved *E*. *coli* proteins [4]. Integrated peak areas (underlined) are provided below the *m/z*. As can be observed, absolute and relative protein ion abundance varies with antibiotic concentration. The B-subunit of Shiga toxin 2 is detected at *m/z* 7821 (middle and bottom panels) and had been previously identified for this strain [27]. Protein ions marked with an asterisk are MALDI matrix adducts. Protein ions marked with a star were analyzed by MS/MS. Protein ions marked with a X were analyzed by MS/MS, but the data was not of sufficient quality for an identification. The protein ion at *m/z* 10410 (bottom panel) highlighted with a question mark resulted in quality MS/MS data (not shown) but was not identified perhaps due to its *in silico* sequence being absent or inaccurate in the genomes that were searched.

**Fig 9:**
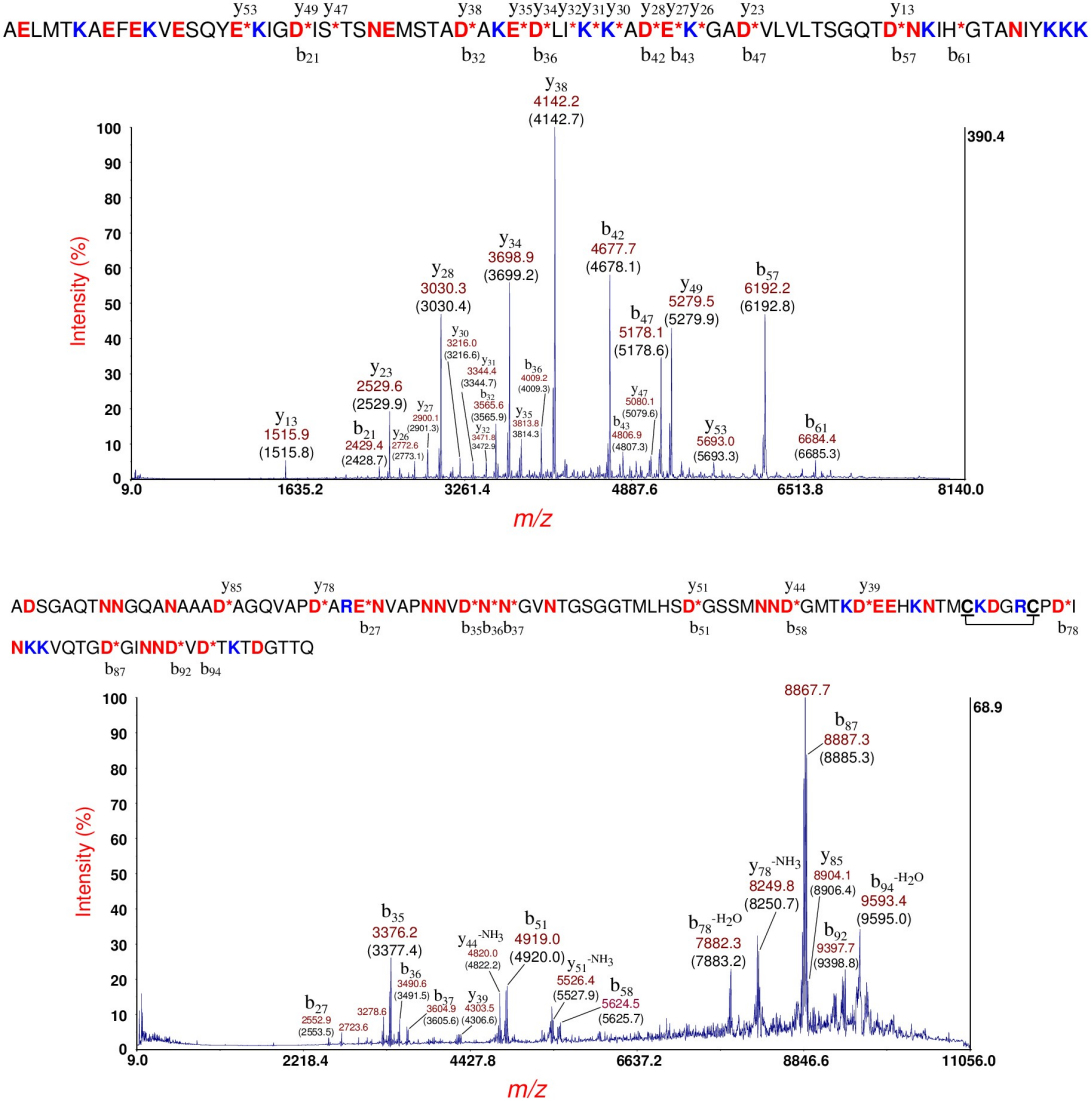
Top panel: MS/MS of the putative YahO protein ion at *m/z* 7708 [M+H]^+^ (Fig 8, top panel). The mature YahO sequence without its signal peptide is shown above. Bottom panel: MS/MS of the putative YbgS protein at *m/z* 10469 [M+H]^+^ (Fig 7, top panel). The protein sequence identification is shown above: YbgS-like protein, hypothetical protein (O3K_17905, AFS75454.1). A 24-residue signal peptide has been removed in the mature sequence. An intramolecular disulfide bond is symbolized by a connecting line between two C-residues (underlined).

Fig 9 (top panel) shows MS/MS of the putative YahO protein ion at *m/z* 7708 [M+H]^+^ (Fig 8, top panel). YahO fragments with high efficiency resulting in numerous fragment ions including seven CFIP. The mature YahO sequence is shown above the spectra. A b- and y-type fragment ion that appear below and above a cleavage site, respectively, constitute a CFIP. The mature YahO sequence has no arginine residues, and it appears that the ionizing proton is distributed randomly amongst the eleven K-residues. YahO has a 21-residue N-terminal signal peptide which is removed post-translationally. The sequence truncation algorithm of our in-house software was found to correctly identify the truncated sequence of the mature YahO protein. The YahO signal peptide cleavage site observed was also consistent with that predicted by SignalP (Version 5.0) [45, 46].

Fig 9 (bottom panel) shows MS/MS of the putative YbgS protein at *m/z* 10469 [M+H]^+^ (Fig 8, top panel). The protein identification is shown above the spectra based on the software identification and manual confirmation: YbgS-like protein [hypothetical protein O3K_17905, AFS75454.1]. The fragmentation efficiency of YbgS is significantly less than that of YahO and there are more small neutral dissociative losses (e.g. loss of NH_3_) due to the presence of two R-residues in the sequence (R26 and R75), and all fragment ions possess one or both R-residues in their sequence. This protein appears to have an intramolecular disulfide bond which is symbolized by a connecting line between two underlined C-residues (C71 and C76). However, we do not detect any fragment ions from PBC within this small-looped structure even though a D73 is present in the loop. For a linear chain protein structure, we would expect PBC on the C-terminal side of D73 producing a b-type fragment ion at *m/z* 7373.6 and/or a y-type fragment ion at *m/z* 3091.3. However, neither of these fragment ions are detected. This suggests that an intramolecular disulfide bond is intact, and it is likely restricting the rotational-vibrational motion necessary of the molecular rearrangement that would precede PBC at D73. Alternatively, the PBC may have occurred at D73 but without breakage of the disulfide bond, however such a scenario would seem unlikely. The sequence truncation algorithm of our in-house software correctly identified the truncated protein sequence of the mature YbgS protein corresponding to removal of a 24-residue N-terminal signal peptide. The signal peptide cleavage site observed was consistent with that predicted by SignalP.

**Fig 10:**
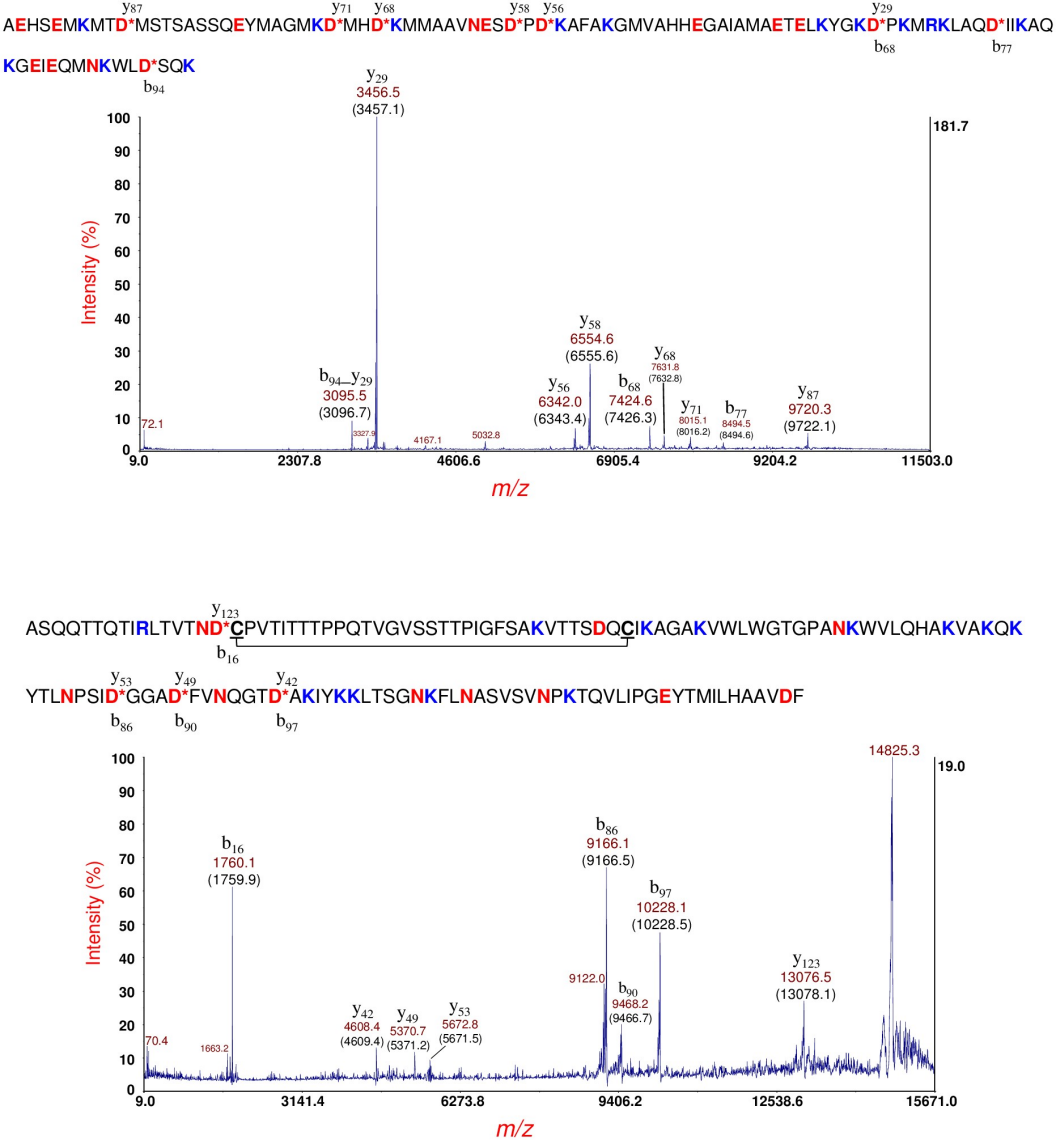
Top panel: MS/MS of the protein ion at *m/z* 10892 [M+H]^+^ shown in Fig 8 (bottom panel). A hypothetical protein (EGT69515.1, C22711_3545) sequence (shown above) was identified. Bottom panel: MS/MS of a protein ion detected at *m/z* 14839 [M+H]^+^ of this strain cultured overnight on LBA supplemented with 60 ng/mL ciprofloxacin. [27] This protein ion also appears at *m/z* 14852 [M+H]^+^ in Fig 8 (bottom panel). The protein was identified as a hypothetical protein (shown above) whose gene is encoded by a plasmid: O3K_26197 (AFS77026.1). A 28-residue N-terminal signal peptide has been removed and there are two cysteine residues: C17 and C49 (underlined), that form an intramolecular disulfide bond (symbolized by a connecting line).

Fig 10 (top panel) shows MS/MS of the protein ion at *m/z* 10892 [M+H]^+^ shown in Fig 8, bottom panel (also detected at *m/z* 10889 and 10894 in the top and middle panels of Fig 8, respectively). A hypothetical protein (EGT69515.1, C22711_3545) was identified from the prominent MS/MS fragment ions. Although this hypothetical protein is of unknown function, it was observed to have a 19-residue N-terminal signal peptide. Interestingly, SignalP predicted a 20-residue N-terminal signal peptide although the probability for the predicted cleavage site was 0.5566. The mature protein sequence of this hypothetical protein has thirteen lysine residues and a single R-residue (R72). All fragment ions except b_68_ have R72 which strongly suggests that the ionizing proton is sequestered primarily, although not exclusively, at R72. Only one CFIP is observed: b_68_/y_29_. Calculation of the protein mass from CFIP gives 3456.5 + 7424.6–2 = 10879.1 Da which is closer to its average theoretical value of 10881.4 Da than that obtained by the MS linear-mode of measurement of 10891 Da. The fragment ion at *m/z* 3095.5 identified as b_94__y_29_ appears to be the result of a dual cleavage on the C-terminal side of D68 followed by D94. Although b_94_ is not detected, it is assumed, given its abundance, that y_29_ is formed initially followed by a second cleavage of the shorter protein fragment on the C-terminal side of D94. Note that this dual cleavage fragment ion retains the most probable site for ionization: R72.

In data not reported previously, Fig 10 (bottom panel) shows MS/MS of a protein ion detected at *m/z* 14839 [M+H]^+^ produced from this strain cultured overnight on LBA supplemented with 60 ng/mL ciprofloxacin [27]. This protein ion also appears at *m/z* 14852 [M+H]^+^ in Fig 8 (bottom panel). The difference in protein ion *m/z* is due to differences in instrument calibration on different days as MS linear-mode has an accuracy of 1000 ppm for external calibration. Analysis of this MS/MS data resulted in the identification of a hypothetical protein whose gene is encoded in a plasmid: O3K_26197 (AFS77026.1). Our software indicates that this protein has a 28-residue N-terminal signal peptide that is removed in the mature protein. The protein sequence has two cysteine residues: C17 and C49 (underlined) that form an intramolecular disulfide bond (symbolized by a connecting line). The sequence of this protein is shown above the MS/MS spectrum, and although the mature protein sequence of this protein has twelve lysine residues, most of the abundant fragment ions include the only R-residue (R10) in their sequence suggesting that R10 is the most probable (although not exclusive) ionization site. Eight prominent fragment ions resulting from PBC on the C-terminal side of four D-residues are detected. These fragment ions comprise four CFIP which allows a more accurate measurement of the mass of the mature protein than that obtained in MS linear-mode. Using the *m/z* of the four CFIP, we obtain a mass of 14835.7 ± 1.4 Da. The theoretical average mass of this protein sequence without its signal peptide and with an intramolecular disulfide bond is 14835.7 Da. The agreement is quite good and better than the MS linear-mode measurement of 14838 Da [27] or 14851 Da (Fig 8, bottom panel). The intramolecular disulfide bond is confirmed both by the mass of the protein as well as by the *absence* of PBC on the C-terminal side of D47 residue which is close to the C49 residue that forms a disulfide bond. It is likely that the molecular rearrangement of the aspartic acid effect is less favored when a D-residue is close to a disulfide bond. We used SignalP to analyze the full protein sequence of this hypothetical protein. SignalP predicted a 28-residue signal peptide consistent with our observed results and supports the simple algorithm used by our software to identify truncated protein sequences. Finally, we would like to note that this protein ion, thus far, is the largest intact protein ion that we have been able to fragment by MS/MS-PSD and identify by top-down analysis. Our results suggest that it is not simply the size of the protein or the number and location of D-residues in its sequence that determines fragmentation efficiency but the degree to which the protein is unfolded *prior* to desorption/ionization. A summary of identifications for *E*. *coli* O104:H4 strain RM14735 is provided in Table 1.

### Identification of bacterial proteins whose genes are induced by antibiotic exposure

Mitomycin-C (MMC) and ciprofloxacin are antibiotics that are commonly used to elicit the SOS response in bacteria and have been shown to be particularly effective at inducing the production of Shiga toxin in many (although not all) STEC strains [26, 27, 47, 48]. The antimicrobial mode-of-action of these antibiotics is damage of bacterial DNA by cross-linking or otherwise interfering with DNA replication. Damage of bacterial DNA can trigger the SOS response which results in the expression of bacteriophage and/or plasmid genes carried by the bacterial host. In order detect and identify proteins produced under antibiotic stress, a sub-inhibitory level of antibiotics is established during culturing on growth media supplemented with the antibiotic. As each bacterial strain is unique in response to an antibiotic, each strain was tested at a variety of concentrations of each antibiotic to detect growth inhibition and yet allow a sufficient number of colonies for harvesting and protein extraction. Fig 2 (top, middle, and bottom panels) show the proteins detected when strain RM7806 was grown on Luria-Bertani agar (LBA) with no antibiotic, LBA supplemented with 400 ng/mL of MMC and LBA supplemented with 600 ng/mL of MMC, respectively. Although this is not a quantitative study, we observe changes in the proteins detected as a function of antibiotic concentration.

Many of the proteins identified appear to be induced by antibiotic exposure, e.g., cold-shock proteins CspC and CspE. These proteins are absent or expressed at undetectable levels when cultured without an antibiotic. For strains RM7806 and RM7807, we observe the production of the immunity cognate protein of antibacterial proteins (colicin E3 and bacteriocin) whose genes are encoded in a plasmid. Previous analysis of this plasmid in a closely related strain (RM7788) revealed a promoter (SOS box) upstream of these genes that, under normal circumstances, is repressed [35]. Upon DNA damage, the repressor (LexA) undergoes self-cleavage allowing expression of genes downstream including bacteriocin and colicin E3 as well as their immunity cognate proteins. We had previously identified expression of Shiga toxin gene (*stx*) from *E*. *coli* O104:H4 strain RM14735 (linked to the 2011 STEC outbreak in Europe) by antibiotic induction and MALDI-TOF-TOF analysis [27]. The *stx* gene is located within a bacteriophage genome which itself is in the bacterial genome. Interestingly, this strain also appears to have inducible plasmid genes.

## Conclusions

We have identified fourteen protein biomarkers from three pathogenic *E*. *coli* strains using antibiotic induction, MALDI-TOF-TOF-MS/MS and top-down proteomic analysis. Mature, intact protein ions were fragmented by PSD and fragment ions formed are the result of aspartic acid effect wherein PBC occurs on the C-terminal side of aspartic acid, glutamic acid, and asparagine residues. Coupled with the measured mass of the biomarker, this well-known fragmentation mechanism results in a characteristic MS/MS profile of high specificity. Both conserved host proteins and plasmid proteins were identified. The protein sequence truncation algorithm of our in-house software used for initial top-down identification successfully identified full protein sequences as well those with a truncated sequence. Observed sequence truncations were consistent with those predicted by SignalP with only one exception in which SignalP mis-identified the signal peptide length of a hypothetical protein by one residue. A distinct trend was observed in the proteins detected with respect to antibiotic concentration. Cold-shock, phage and plasmid proteins increased with antibiotic concentration whereas acid-stress proteins (and other conserved proteins) decreased. Of particular interest was the detection of HPr, a global regulator carbon utilization, caused by antibiotic exposure.

## Supporting information

S1 Fig MS of sample supernatant of *E*. *coli* O113:H21 strain RM7806 cultured overnight on LBA supplemented with 400 ng/mL of MMC. Protein ion at *m/z* 9779 (marked with star) was analyzed by MS/MS (Fig 4, bottom panel).
(PPTX)

S2 Fig MS of sample supernatant of *E*. *coli* O113:H21 strain RM7807 cultured overnight on LBA supplemented with 400 ng/mL of MMC. Insert shows an expanded *m/z* region. Protein ions labeled HdeA and HdeB are highly conserved acid stress proteins previously identified in other *E*. *coli* strains. Protein ion at *m/z* 9778 (marked with star) was analyzed by MS/MS (Fig 7, middle panel).
(PPTX)
