# Supplementary figures and images for: Top-down proteomic identification of plasmid and host proteins produced by pathogenic Escherichia coli using MALDI-TOF-TOF tandem mass spectrometry
Source: PLoS One. 2021 Nov 29;16(11):e0260650. doi: 10.1371/journal.pone.0260650 (PMC8629258; doi:10.1371/journal.pone.0260650)

## Slide 1
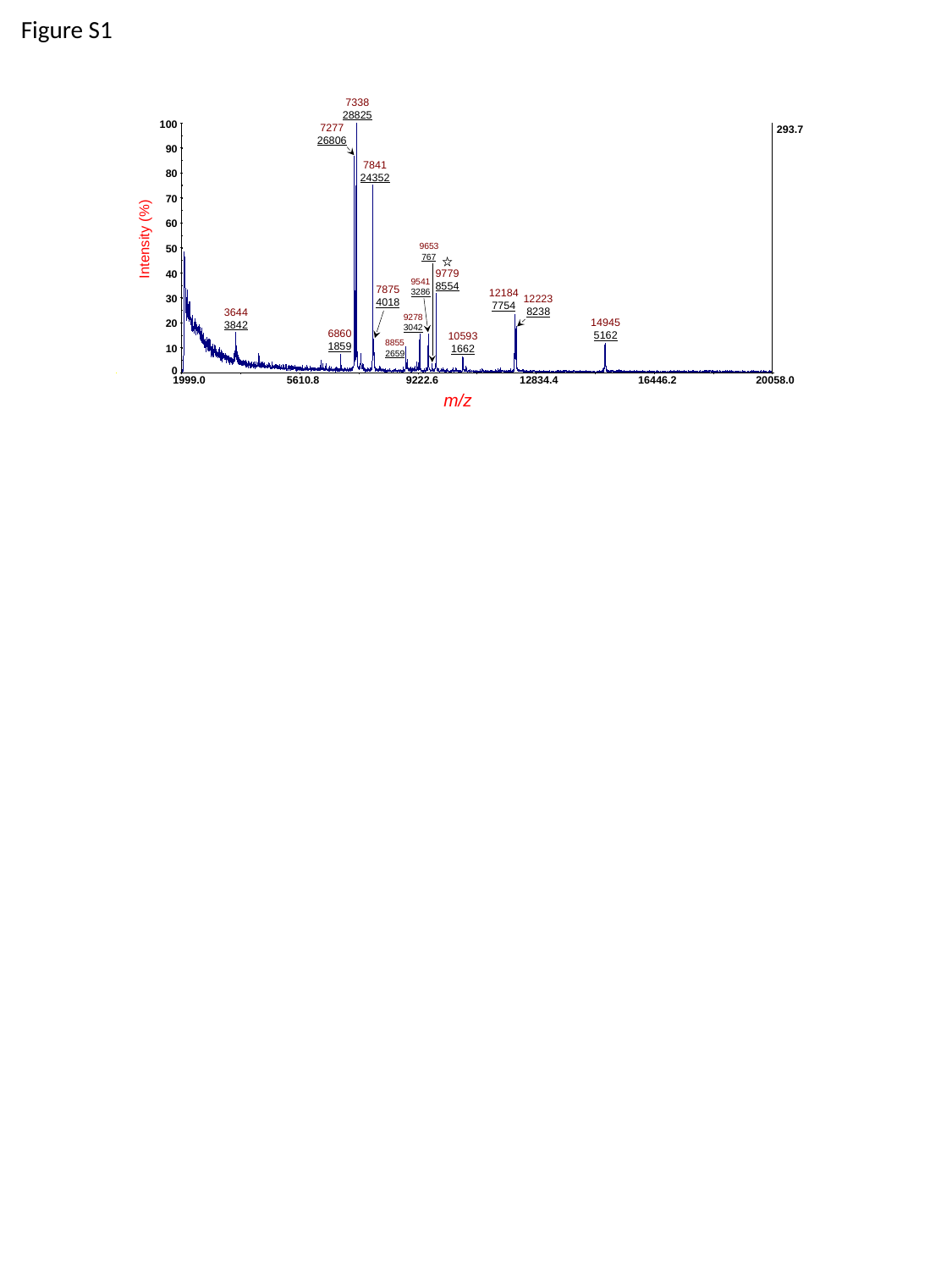

Figure S1
7338
28825
100
7277
26806
293.7
90
7841
24352
80
70
60
Intensity (%)
9653
767
50
9779
8554
40
9541
3286
7875
4018
12184
7754
30
12223
8238
3644
3842
9278
3042
14945
5162
20
6860
1859
10593
1662
8855
2659
10
0
1999.0
5610.8
9222.6
12834.4
16446.2
20058.0
m/z

Supplement: S1 Fig — Protein ion at m/z 9779 (marked with star) was analyzed by MS/MS (Fig 4, bottom panel). (PPTX) [file pone.0260650.s001.pptx]

## Slide 1
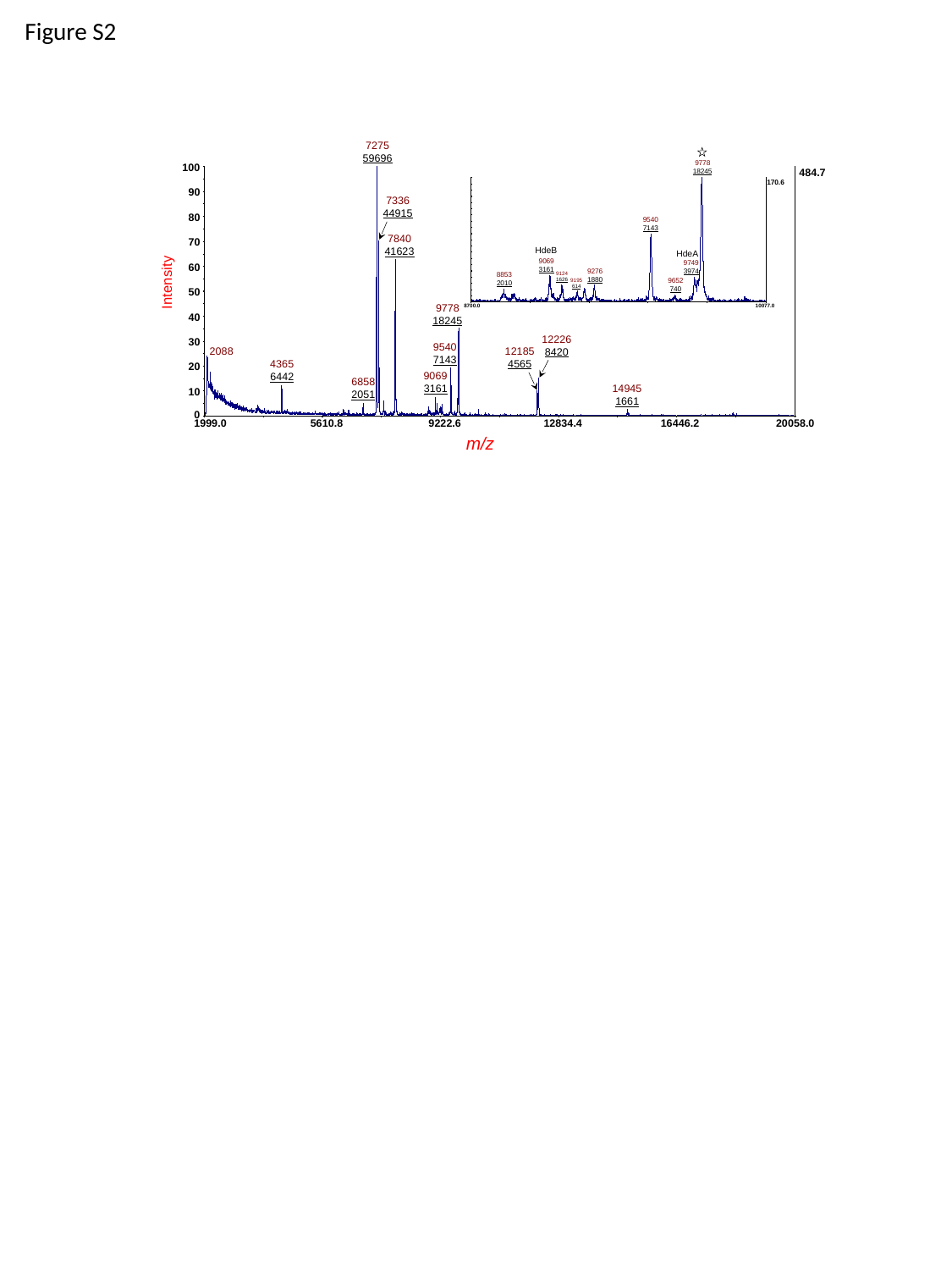

Figure S2
7275
59696
100
484.7
90
7336
44915
80
7840
41623
70
60
Intensity
50
9778
18245
40
12226
8420
30
9540
7143
12185
4565
2088
4365
6442
20
9069
3161
6858
2051
14945
1661
10
0
1999.0
5610.8
9222.6
12834.4
16446.2
20058.0
m/z
9778
18245
170.6
9540
7143
9069
3161
9749
3974
9276
1880
9124
1626
8853
2010
9652
740
9195
614
8700.0
10077.0
HdeB
HdeA

Supplement: S2 Fig — Insert shows an expanded m/z region. Protein ions labeled HdeA and HdeB are highly conserved acid stress proteins previously identified in other E. coli strains. Protein ion at m/z 9778 (marked with star) was analyzed by MS/MS (Fig 7, middle panel). (PPTX) [file pone.0260650.s002.pptx]
